# Importance of ELABELA in the differential diagnosis of benign and malignant lesions of the thyroid gland

**DOI:** 10.20945/2359-3997000000293

**Published:** 2020-10-09

**Authors:** Mehmet Bankir, Cansu Abayli, Fettah Acibucu

**Affiliations:** 1 University of Health Sciences Adana City Training and Research Hospital Department of Internal Medicine Adana Turkey Department of Internal Medicine, University of Health Sciences – Adana City Training and Research Hospital, Adana, Turkey; 2 University of Health Sciences Adana City Training and Research Hospital Department of Pathology Adana Turkey Department of Pathology, University of Health Sciences – Adana City Training and Research Hospital, Adana, Turkey; 3 University of Health Sciences Adana City Training and Research Hospital Department of Internal Medicine Adana Turkey Department of Internal Medicine, Endocrinology Division, University of Health Sciences – Adana City Training and Research Hospital, Adana, Turkey

**Keywords:** Apelin, APJ, thyroid, toddler

## Abstract

**Objective:**

This study investigated whether ELABELA plays a role in the differential diagnosis of benign and malignant lesions of the thyroid gland.

**Subjects and methods:**

Of the 87 patients included in the study, 12 had undergone surgery for benign thyroid diseases, 30 had papillary thyroid cancer without invasion and/or lymph node metastasis in the surrounding tissues in the pathology report, and 45 had papillary thyroid cancer with invasion and/or lymph node metastasis in the surrounding tissues.

**Results:**

In the macrocarcinoma group, the proportion of patients with severe ELABELA staining (61.1%) was higher than that in the adenoma (50%) and microcarcinoma (23.8%) groups, while the proportion of those with mild to moderate staining was lower (p < 0.001). In the microcarcinoma group, the proportion of patients with severe staining was lower than that in the adenoma group, while the proportion of those with mild to moderate staining was higher (p < 0.001). In papillary thyroid carcinomas, the rates of moderate and severe staining in the classical variant, mild staining in the follicular variant, severe staining in the classical + follicular variant, and severe staining in the oncocytic variant were higher.

**Conclusion:**

To the best of our knowledge, this study is the first to be conducted on this subject. In this study, ELABELA was not found to be significant in the differential diagnosis of benign and malignant lesions of the thyroid gland. In papillary thyroid carcinomas, severe ELABELA staining patterns were more common in macrocarcinoma patients than in microcarcinoma patients.

## INTRODUCTION

Although thyroid cancers are rare among all cancers, they are most common among cancers of endocrine organs ( 1 ). Thyroid cancer has not been widespread in the past, but its rates are increasing, likely related to new technological advancements that make it possible to detect small thyroid cancers ( 2 ).

Thyroid cancers are categorized into three groups. The most frequently observed type of thyroid cancer is papillary thyroid cancer, with a rate of 85%. Follicular cancers follow with a rate of 12%, while undifferentiated cancers constitute 3% of thyroid cancers ( 3 ). Laboratory examinations, thyroid ultrasonography, thyroid scintigraphy, and pathological methods are the most frequently used methods in the differential diagnosis of thyroid cancers ( 4 – 6 ). The definitive diagnosis method remains a tissue diagnosis with a pathological diagnosis.

Pathologically, cells extracted from thyroid lesions are stained with haematoxylin and eosin after preparation under appropriate conditions ( 7 ). The immunohistochemical expression levels of molecules, such as HBME-1, AE1/AE3, BRAF V600E, galectin 3, CK19, and CD56, are then examined to perform the differential diagnosis of thyroid cancer ( 8 ). ELABELA is a molecule that has been used recently in the diagnosis of some malignant cells.

ELABELA is an endogenic peptide ligand of the apelin receptor ( 9 ) that is expressed in human pluripotent stem cells ( 10 ). Therefore, it functions both during and after embryonic development. ELABELA is a hormone that demonstrates both paracrine and endocrine functions ( 11 ). In recent studies, ELABELA demonstrated immunoreactivity in kidney cell tumours and gliomas ( 12 , 13 ).

ELABELA can be used in the diagnosis of malignancies of other hormonal cells because it is expressed in both embryonic and pluripotent stem cells and because it is a hormone that can be used for the immunohistochemical staining of malignant cells.

Therefore, we investigated whether ELABELA plays a role in the differential diagnosis of benign and malignant lesions of the thyroid gland.

## SUBJECTS AND METHODS

### Study population

This retrospective study was conducted between December 15, 2017 and June 15, 2018 at Adana City Hospital's Internal Diseases Clinic. Ethics committee approval was obtained from the Adana City Hospital Ethics Committee (date: 07.12.2018; decision number: 40). Informed consent was obtained from the participants included in the study.

Eighty-seven patients who were followed up at our clinic between 2015 and 2018 for thyroid diseases were included in the study. Of the 87 included patients, 12 had undergone surgery for benign thyroid diseases, 30 had papillary thyroid cancer without invasion and/or lymph node metastasis in the surrounding tissues in the pathology report, and 45 had papillary thyroid cancer with invasion and/or lymph node metastasis in the surrounding tissues. Participants between the ages of 18 and 85 years were included in the study according to file order without sex discrimination.

Patients with known diabetes mellitus, hypertension, acute or chronic renal failure, cerebrovascular accidents, coronary artery disease, nephrotic syndrome, acute or chronic liver failure, endocrine hormonal disorders, and any malignant diagnosis were excluded from the study.

All patients included in the study were diagnosed after laboratory, radiological, and pathological examinations.

Thyroid papillary microcarcinoma is a subtype of papillary carcinoma that includes tumours less than 10 mm in diameter; if the tumour size is greater than 1 cm, it is defined as a macrocarcinoma.

### Immunohistochemical staining of ELABELA

**Figure 1:**
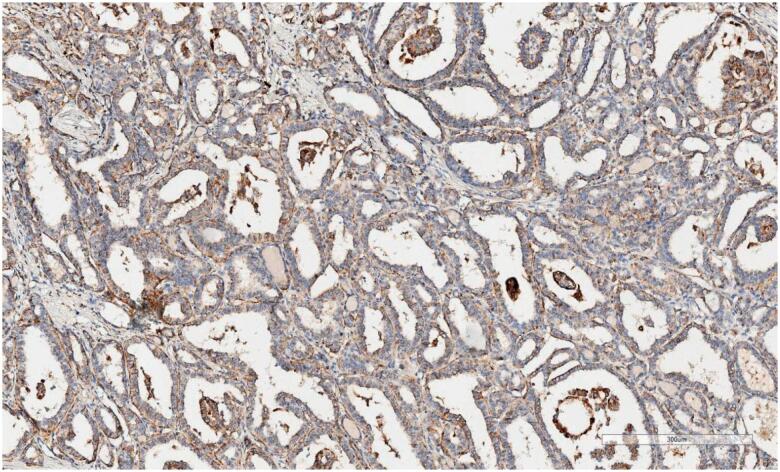
Mild degree of stoplasmic and membranous staining.

**Figure 2:**
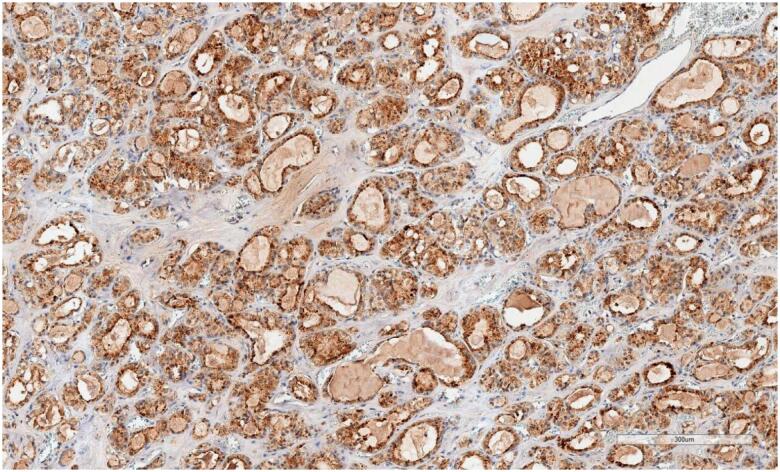
Moderate staining.

**Figure 3:**
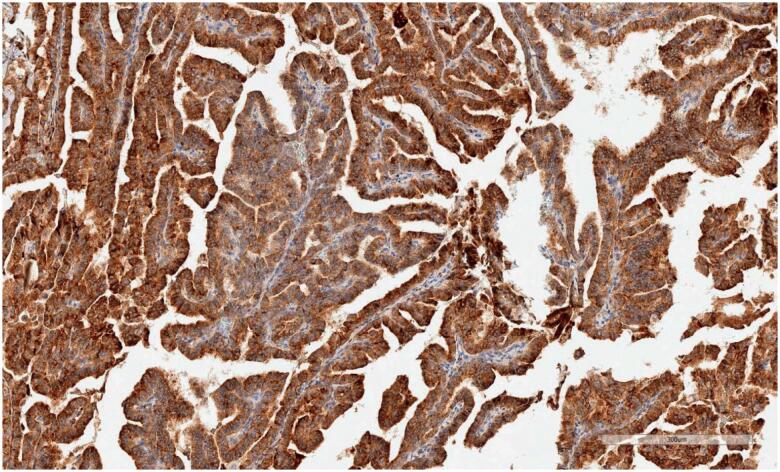
Severe degree of staining.

Blocks allocated for apelin study were prepared by making 3-µm-thick sections on positively charged slides. A deparaffinisation process was performed on the device after 40 min in an oven at 60 °C. Apelin antibody (2A1-2D5), prepared at a 1:100 ratio, was processed in Leica Bond Max devices. Photography was performed using an Aperio CS2 Leica device (0: no staining, +1: mild staining, +2: moderate, +3: severe). ELABELA staining was scored as follows: +1: mild degree of stoplasmic and membranous staining ( Figure 1 ), +2: moderate ( Figure 2 ), +3: severe degree of staining ( Figure 3 ).

### Statistical analysis

Statistical analysis was conducted using SPSS 20 for Windows (IBM SPSS Inc., Armonk, NY, USA). Normal distribution of the data was analysed using the Kolmogorov-Smirnov test. Normally distributed numerical variables were shown as means ± standard deviation, while non-normally distributed numerical variables were shown as medians (min-max). Categorical variables were shown as numbers and percentages. The comparison between nominal variables was made by Chi-squared analysis. In cases where the expected values in the 2×2 tables do not have a sufficient volume, Fisher's exact test was used and Monte Carlo Simulation was applied in the R×C tables. The significant difference in the numerical variables between the two groups was evaluated by unpaired t-test (normally distributed numerical variables) and the Mann-Whitney *U* -test (non-normally distributed numerical variables). The significant difference of the numerical variables among the three groups was analysed by ANOVA (post hoc: Bonferroni test) (normally distributed numerical variables) and the Kruskal-Wallis H test (post hoc: Dunn's test) (non-normally distributed numerical variables). Statistical differences were marked in bold (for post hoc: p value < 0.05).

A p-value < 0.05 was accepted as significant in the statistical analyses.

## RESULTS

The clinical and demographic findings of the study population are shown in Table 1 in detail. The mean age of the study population was 48 ± 15.3 years, and 13.8% of the patients (n = 12) had adenoma while 86.2% of the patients (n = 75) had thyroid carcinoma. The mean age showed no significant difference in patients with thyroid carcinoma compared with patients with adenoma. The proportion of women with thyroid carcinoma was higher than that of women with adenoma (82.7% vs. 50%, respectively; p = 0.030). Furthermore, 28% (n: 21) of the patients with thyroid carcinoma had microcarcinoma while 72% (n = 54) had macrocarcinoma. The distribution of the histological variants among the patients with thyroid carcinoma were as follows: classical variant, 70.7% (n = 53); follicular variant, 26.7% (n = 20); classical + follicular variant, 1.3% (n = 1), and oncocytic variant for 1.3% (n = 1). The median tumour diameter was lower in thyroid carcinoma patients than in the adenoma group (12 vs. 40, respectively; p < 0.001). Capsular invasion was found in 17.3% (n = 13) of the patients with thyroid carcinoma, in 18.7% (n = 14) of the patients with lymphovascular invasion perineural invasion in 1.3% (n = 1), and in 6.7% (n = 5) of patients with extrathyroidal invasion. No patient with capsular, lymphovascular, perineural, or extrathyroidal invasion was found in the adenoma group. In the adenoma group, severe staining was found in 50% of tissues (n = 6) by ELABELA staining, moderate staining was found in 41.7% (n = 5), and mild staining was found in 8.3% (n = 1). In the thyroid carcinoma group, severe staining was found in 50.7% of the tissues (n = 38) by ELABELA staining, moderate staining was found in 37.3% (n = 28), and mild staining was found in 12% (n = 9). No significant difference was found between the severity of ELABELA staining in the thyroid and adenoma groups (p = 0.913).

**Table 1:** Demographic and clinical distributions

Variables		Total population n = 87	Adenoma n = 12	Thyroid cCarcinoma n = 75	p
Age (years)		48.0 ± 15.3	47.8 ± 15.6	48.1 ± 15.4	0.945
Gender, n (%)					
	Female	68 (78.2)	6 (50.0)	62 (82.7)	
	Male	19 (21.8)	6 (50.0)	13 (17.3)	
Tumor diameter (mm)		13 (0.5-80)	40 (20-80)	12 (0.5-80)	<0.001 *
Capsular invasion, n (%)		13 (14.9)	−	13 (17.3)	−
Lymphovascular invasion, n (%)		14 (16.1)	−	14 (18.7)	−
Perineural invasion, n (%)		1 (1.1)	−	1 (1.3)	−
Extrathyroidal invasion, n (%)		5 (5.7)	−	5 (6.7)	−
Invasive tissue invasion, n (%)		11 (14.7)	−	11 (14.7)	−
Multiple tumor focus, n (%)		21 (28.0)	−	21 (28.0)	−
ELABELA staining, n (%)					
	Mild (+)	10 (11.5)	1 (8.3)	9 (12.0)	
	Middle (++)	33 (37.9)	5 (41.7)	28 (37.3)	0.913
	Severe (+++)	44 (50.6)	6 (50.0)	38 (50.7)	

Categorical variables were expressed as numbers (%).

Numerical variables were expressed as mean ± standard deviation or median (min-max).

* p <0.05 shows statistical significance.

No significant difference was observed between the mean ages of the microcarcinoma, macrocarcinoma, and adenoma groups. The number of women in the microcarcinoma group was higher than that in the other groups. The number of women in the macrocarcinoma group was also higher than that in the adenoma group (p = 0.048). No significant difference was found in the histological variant distribution between the microcarcinoma and macrocarcinoma groups (p=0.218). The median tumour diameter in the adenoma group was higher than that in the microcarcinoma and macrocarcinoma groups (p < 0.001). No significant difference was found in the rates of capsular, lymphovascular, perineural, and extrathyroidal invasions between the microcarcinoma and macrocarcinoma groups. The rate of patients with severe ELABELA staining in the macrocarcinoma group (61.1%) was higher than that in the adenoma (50%) and microcarcinoma (23.8%) groups, while the rates of patients with mild and moderate staining were lower (p < 0.001). The rate of patients with severe ELABELA staining in the microcarcinoma group was lower than that in the adenoma group, while the rates of patients with mild and moderate staining were also lower (p < 0.001) ( Table 2 ).

**Table 2:** Demographic and clinical distributions by histological subtypes

Variables		Adenoma n = 12	Microcarcinoma n = 21	Macrocarcinoma n = 54	p
Age (years)					
Gender, n (%)		47.8 ± 15.6	49.2 ± 15	47.6 ± 15.6	0.920
	Female	6 (50.0)	18 (85.7)	44 (81.5)	0.048 *
	Male	6 (50.0)	3 (14.3)	10 (18.5)	
Histological variant					
	Classic variant	−	12 (57.1)	41 (75.9)	
	Follicular variant	−	9 (42.9)	11 (20.4)	
	Classic + follicular variant	−	−	1 (1.9)	
	Oncositic variant	−	−	1 (1.9)	0.218
**Tumor diameter (mm)**		**40 (20-80)**	**7 (0.5-13)**	**15 (4-80)**	<0.001 *
Capsular invasion, n (%)		−	1 (4.8)	12 (22.2)	0.146
Lymphovascular invasion, n (%)		−	2 (9.5)	12 (22.2)	0.349
Perineural invasion, n (%)		−	−	1 (1.9)	0.999
Extrathyroidal invasion, n (%)		−	1 (4.8)	4 (7.4)	0.999
Invasive tissue invasion, n (%)		−	1 (4.8)	10 (18.5)	0.251
Multiple tumor focus, n (%)		−	5 (23.8)	16 (29.6)	0.777
ELABELA staining, n (%)					
Mild (+)		1 (8.3)	7 (33.3)	2 (3.7)	
Middle (++)		5 (41.7)	9 (42.9)	19 (35.2)	
Severe (+++)		6 (50.0)	5 (23.8)	33 (61.1)	

Categorical variables were expressed as numbers (%).

Numerical variables were expressed as mean ± standard deviation or median (min-max).

* p <0.05 shows statistical significance.

Bold characters indicate differences between groups (posthoc: Dun's test p<0.05).

In the microcarcinoma patients, no significant difference was found between the demographic and clinical findings of the groups with moderate and severe staining. Among the macrocarcinoma patients, no significant difference was found between the demographic and clinical findings of the groups with moderate and severe staining ( Table 3 ).

**Table 3:** Demographic and clinical distributions of histological subtypes according to Elabela severity

Variables		Microcarcinoma		p	Microcarcinoma		p
		Middle (++) n = 9	Severe (+++) n = 5		Middle (++) n = 19	Severe (+++) n = 33	
Age (years)		45.6 ± 12.2	50.6 ± 11.3	0.462	45.5 ± 13.9	48.5 ± 16.9	0.517
Gender, n (%)							
	Female	8 (88.9)	4 (80.0)	0.999	16 (84.2)	26 (78.8)	0.910
	Male	1 (11.1)	1 (20.0)		3 (15.8)	7 (21.2)	
Histological variant, n(%)							
	Classic	7 (77.8)	3 (60.0)	16 (84.2)	24 (72.7)		
	Follicular	2 (22.2)	2 (40.0)	3 (15.8)	7 (21.2)		
	Classic + follicular	−	−		−	1 (3.0)	0.891
	Oncositic	−	−		−	1 (3.0)	
Tumor diameter (mm)		7 (3-13)	8 (2-9)	0.898	13 (7-45)	16 (11-80)	0.134
Capsular invasion, n (%)		−	−	−	4 (21.1)	8 (24.2)	0.999
Lymphovascular invasion, n (%)		1 (11.1)	−	0.999	5 (26.3)	7 (21.2)	0.937
Perineural invasion, n (%)		−	−	−	1 (5.3)	−	0.778
Extrathyroidal invasion, n (%)		1 (11.1)	−	0.999	1 (5.3)	3 (9.1)	0.999
Invasive tissue invasion, n (%)		−	−	−	4 (21.1)	6 (18.2)	0.999
Multiple tumor focus, n (%)		2 (22.2)	1 (20.0)	0.999	6 (31.6)	9 (27.3)	0.990

Categorical variables were expressed as numbers (%).

Numerical variables were expressed as mean ± standard deviation median (min-max).

* p <0.05 shows statistical significance.

## DISCUSSION

In our study, the rate of severe staining with ELABELA was higher in the macrocarcinoma group than in the microcarcinoma and adenoma groups. In papillary thyroid carcinomas, the rates of moderate and severe staining were higher in the classical variant, mild staining was found in the follicular variant, severe staining was found in the classical + follicular variant, and severe staining was observed in the oncocytic variant. To the best of our knowledge, this study is the first to be conducted on this subject.

ELABELA is a recently discovered endogenous peptide ligand of the apelin receptor ( 14 ). Although ELABELA is associated with zebra fish, high preservation of the ELABELA gene occurs in vertebrate species, including humans, leading to the opinion that it is an important molecule in human development ( 15 ). ELABELA is a natural hormone that functions both during and after the embryonic development process. Studies conducted to date have shown the expression of both the apelin receptor and apelin in the brain, heart, kidneys, lungs, and other vascular structures ( 16 – 18 ). Similarly, apelin plays an important role in various biological functions, such as homeostasis and fluid metabolism of apelinergic and cardiovascular systems ( 19 , 20 ). Apelin is an endogenous ligand of isoforms, such as apelin-13, apelin-17, and apelin-36, along with the G-protein-coupled receptor ( 21 ). We believe that ELABELA is associated with many systems and tissues because it is a hormone found in both embryonic stem cells and pluripotent stem cells. However, the literature data on this topic are limited. We have only encountered two studies in the literature including patients with malignancies ( 12 , 13 ).

Artas and cols. investigated whether ELABELA plays a role in benign and malignant renal tumours ( 13 ). ELABELA reactivity was lower in renal oncocytomas than in the control group, and ELABELA immunoreactivity was lower in carcinomas with chromophobe renal cells than in the control group. Additionally, they found no ELABELA immunoreactivity in carcinomas with papillary renal cells, and ELABELA immunoreactivity was higher in oncocytomas, which are benign kidney tumours. Furthermore, Artas and cols. ( 12 ) examined whether ELABELA plays a role in the pathological grading of gliomas. In that study, ELABELA immunoreactivity was higher in glioma tissues than in normal brain tissues. In high-grade gliomas, ELABELA immunoreactivity was higher than that in low-grade gliomas, and ELABELA histoscores were found to be respectively higher than those of low-grade glioma and normal brain tissue. We could not find any other studies conducted on this subject.

In our study, we examined the role of ELABELA in benign and malignant thyroid tumours. No significant difference was found in either adenomas or thyroid carcinomas regarding the staining patterns of ELABELA. In most cases in these two groups, severe staining was found, followed by moderate and mild staining. However, tissues with macrocarcinoma were more severely stained than those with microcarcinoma and adenoma when we separated papillary thyroid carcinomas as micro- and macrocarcinomas. Furthermore, the severity of staining increased as the size of the tumour grew. No correlation was observed between surrounding tissue invasion or lymphatic invasion of the papillary thyroid carcinomas concerning the ELABELA staining patterns. However, the moderate and severe staining rates were higher in the classical variant, mild staining was found in the follicular variant, and severe staining was found in the classical + follicular and oncocytic variants when we examined the staining patterns of these histological variants.

In the study conducted on kidney tumours by Artas and cols. ( 13 ), ELABELA immunoreactivity was higher in benign tumours. However, in their study conducted on brain tumours ( 12 ), ELABELA immunoreactivity was higher in gliomas than in normal brain tissue. In our study, a benign/malignant differential diagnosis could not be made; in papillary thyroid carcinomas, a more severe staining pattern was found in macrocarcinomas than in microcarcinomas. Thus, ELABELA cannot serve as a marker in the differential diagnosis of benign and malignant tumours in all tissues. ELABELA may be related to different parameters in the staining patterns of benign and malignant cells. In another study, ELABELA was expressed in the brain, heart, kidneys, lungs, and other vascular structures ( 21 ). However, we do not have any data available on whether this molecule is expressed in the thyroid gland. For this purpose, in vivo and in vitro studies are needed.

The main limitation of our study is that it is cross-sectional. Other limitations are the low number of patients, differences in the numbers of patients among the groups, and that 12 patients had benign thyroid lesions. An additional limitation is that histoscores were studied when pathological examination was performed, but the immunoreactivity of ELABELA in the tissues was not examined.

In conclusion, ELABELA was not found to be significant in the differential diagnosis of benign and malignant thyroid tumours. In papillary thyroid carcinomas, the rate of severe ELABELA staining patterns was higher in macrocarcinomas than in microcarcinomas. To determine whether ELABELA is a molecule that can be used in the differential diagnosis of benign and malignant thyroid tumours, extensive studies with both normal thyroid tissue and benign and malignant thyroid tumours as well as participants in higher numbers are needed.

## References

[1] Katoh H, Yamashita K, Enomoto R, Watanabe M. Classification and general considerations of thyroid cancer. Ann Clin Pathol. 2015;3(1):1045.

[2] Brito JP, Morris JC, Montori VM. Thyroid cancer: zealous imaging has increased detection and treatment of low risk tumours. BMJ. 2013;347:f4706. 10.1136/bmj.f4706 23982465

[3] Schlumberger MJ. Papillary and follicular thyroid carcinoma. N Engl J Med. 1998;338(5):297-306. 10.1056/NEJM199801293380506 9445411

[4] Frilling A, Tecklenborg K, Görges R, Weber F, Clausen M, Broelsch EC. Preoperative diagnostic value of [(18)F] fluorodeoxyglucose positron emission tomography in patients with radioiodine-negative recurrent well-differentiated thyroid carcinoma. Ann Surg. 2001;234(6):804-11. 10.1097/00000658-200112000-00012 PMC1422140 11729387

[5] Shimura H, Haraguchi K, Hiejima Y, Fukunari N, Fujimoto Y, Katagiri M, et al. Distinct diagnostic criteria for ultrasonographic examination of papillary thyroid carcinoma: a multicenter study. Thyroid. 2005;15(3):251-8. 10.1089/thy.2005.15.251 15785244

[6] Cooper DS, Doherty GM, Haugen BR, Kloos RT, Lee SL, Mandel SJ, et al.; American Thyroid Association (ATA) Guidelines Taskforce on Thyroid Nodules and Differentiated Thyroid Cancer. Revised American Thyroid Association management guidelines for patients with thyroid nodules and differentiated thyroid cancer. Thyroid. 2009;19(11):1167-214. 10.1089/thy.2009.0110 19860577

[7] Lassalle S, Hofman V, Marius I, Gavric-Tanga V, Brest P, Havet K, et al. Assessment of morphology, antigenicity, and nucleic acid integrity for diagnostic thyroid pathology using formalin substitute fixatives. Thyroid. 2009;19(11):1239-48. 10.1089/thy.2009.0095 19888862

[8] Baloch Z, Mete O, Asa SL. Immunohistochemical biomarkers in thyroid pathology. Endocr Pathol. 2018;29(2):91-112. 10.1007/s12022-018-9532-9 29744727

[9] Yang P, Maguire JJ, Davenport AP. Apelin, Elabela/Toddler, and biased agonists as novel therapeutic agents in the cardiovascular system. Trends Pharmacol Sci. 2015;36(9):560-7. 10.1016/j.tips.2015.06.002 PMC4577653 26143239

[10] Wang Z, Yu D, Wang M, Wang Q, Kouznetsova J, Yang R, et al. Elabela-apelin receptor signaling pathway is functional in mammalian systems. Sci Rep. 2015;5:8170. 10.1038/srep08170 PMC4313117 25639753

[11] Huang R, Zhu J, Zhang L, Hua X, Ye W, Chen C, et al. Is ELABELA a reliable biomarker for hypertensive disorders of pregnancy? Pregnancy Hypertens. 2019;17:226-32. 10.1016/j.preghy.2019.06.007 PMC7001771 31487645

[12] Artas G, Ozturk S, Kuloglu T, Dagli AF, Gonen M, Artas H, et al. A novel candidate molecule in the pathological grading of gliomas: ELABELA. Turk Neurosurg. 2018;28(6):989-94. 10.5137/1019-5149.JTN.22022-17.2 29694663

[13] Artas G, Kuloglu T, Dagli AF, Ugur K, Yardim M, Aydin S, et al. A promising biomarker to distinguish benign and malignant renal tumors: ELABELA. Niger J Clin Pract. 2019;22(3):386-92. 10.4103/njcp.njcp_105_18 30837428

[14] Ho L, van Dijk M, Chye STJ, Messerschmidt DM, Chng SC, Ong S, et al. ELABELA deficiency promotes preeclampsia and cardiovascular malformations in mice. Science. 2017;357(6352):707-13. 10.1126/science.aam6607 28663440

[15] Chng SC, Ho L, Tian J, Reversade B. ELABELA: a hormone essential for heart development signals via the apelin receptor. Dev Cell. 2013;27(6):672-80. 10.1016/j.devcel.2013.11.002 24316148

[16] Kleinz MJ, Skepper JN, Davenport AP. Immunocytochemical localisation of the apelin receptor, APJ, to human cardiomyocytes, vascular smooth muscle and endothelial cells. Regul Pept. 2005;126(3):233-40. 10.1016/j.regpep.2004.10.019 15664671

[17] Chandrasekaran B, Dar O, McDonagh T. The role of apelin in cardiovascular function and heart failure. Eur J Heart Fail. 2008;10(8):725-32. 10.1016/j.ejheart.2008.06.002 18583184

[18] Kleinz MJ, Davenport AP. Immunocytochemical localization of the endogenous vasoactive peptide apelin to human vascular and endocardial endothelial cells. Regul Pept. 2004;118(3):119-25. 10.1016/j.regpep.2003.11.002 15003827

[19] Galanth C, Hus-Citharel A, Li B, Llorens-Cortès C. Apelin in the control of body fluid homeostasis and cardiovascular functions. Curr Pharm Des. 2012;18(6):789-98. 10.2174/138161212799277770 22236125

[20] Medhurst AD, Jennings CA, Robbins MJ, Davis RP, Ellis C, Winborn KY, et al. Pharmacological and immunohistochemical characterization of the APJ receptor and its endogenous ligand apelin. J Neurochem. 2003;84(5):1162-72. 10.1046/j.1471-4159.2003.01587.x 12603839

[21] Chapman NA, Dupré DJ, Rainey JK. The apelin receptor: physiology, pathology, cell signalling, and ligand modulation of a peptide-activated class A GPCR. Biochem Cell Biol. 2014;92(6):431-40. 10.1139/bcb-2014-0072 PMC4894813 25275559

